# Experimental and numerical datasets for benchmark tests on high strength steel I-section frames

**DOI:** 10.1016/j.dib.2024.110049

**Published:** 2024-01-09

**Authors:** Fiona Walport, Yufei Zhu, Xiang Yun, Leroy Gardner

**Affiliations:** a Department of Civil and Environmental Engineering, Imperial College London, London, UK; b School of Civil Engineering, Shanghai Normal University, Shanghai, PR China; c Department of Civil Engineering, University of Bristol, Bristol, UK

**Keywords:** Bending tests, Eurocodes, Finite element modelling, GMNIA, Structural analysis, Validation

## Abstract

This data article presents experimental and numerical datasets for eight fixed-base, single storey, unbraced high strength steel welded I-section frames subjected to in-plane horizontal and vertical loading. A detailed description of the full-scale frame testing programme is provided in the related research article ‘Benchmark tests on high strength steel frames’. The experimental dataset can be used to steer future research in full-scale structural testing and provide benchmark results that are suitable for the validation of finite element models and the development of system-level design approaches. In addition to the benchmark experimental frame data, all necessary details and data for shell finite element (FE) model validation using geometrically and materially nonlinear analysis (GMNIA) is presented. The general purpose FE software Abaqus was used. The dataset can be used as an illustrative example of GMNIA validation in accordance with EN 1993–1–14 with all relevant data for reproducibility provided.

Specifications Table

**Table:** 

Subject	Civil and Structural Engineering
Specific subject area	Structural testing and numerical model validation
Data format	Raw, Analyzed, Filtered
Type of data	Tables, Images, Graphs, Figures, Finite element model input files
Data collection	Test instrumentation: electrical resistance strain gauges, string potentiometers, inclinometers, fabricated load cells. Numerical simulations: Matlab 2020a (generation of input files), Abaqus 2021 (numerical simulations), CUFSM v4.05 (calculation of local imperfection local buckling half-wavelengths). Data curation: Excel Version 2310.
Data source location	• Institution: Imperial College London • City/Town/Region: London • Country: UK
Data accessibility	Repository name: Mendeley Data Data identification number: 10.17632/38xk9wfv7b.1 Direct URL to data: https://data.mendeley.com/datasets/38xk9wfv7b/1
Related research article	X. Yun, Y. Zhu, Z. Wang, L. Gardner, Benchmark tests on high strength steel frames, Eng Struct. 258 (2022) 114108. https://doi.org/10.1016/j.engstruct.2022.114108

## 1 Value of the Data

• This data article presents the experimental data for eight fixed-base, single storey, unbraced high strength steel welded I-section frames subjected to in-plane horizontal and vertical loading [1], as well as the corresponding shell finite element models and numerical simulation data for geometrically and materially nonlinear analysis with imperfections (GMNIA).
• With the development of the upcoming EN 1993–1–14 [2], design by finite element analysis is likely to become more widespread in structural engineering practice. This study acts as a GMNIA validation example consisting of presented laboratory test data and the supporting numerical simulations and all relevant data for reproducibility.
• The experimental data can aid the design of future large-scale experimental programmes, as well as be used as benchmark data for the validation of advanced numerical models and system-level design approaches.
• The finite element models of the imperfect frames can be directly opened in Abaqus and further studied using any of the analyses and graphical visualisation features available in the software. The numerical simulation data can be used and/or reproduced as a basis for comparison with the results of other geometrically and materially nonlinear analyses with imperfection (GMNIA).

## 2 Data Description

This data article presents the data from structural tests on eight fixed-base, single storey, unbraced high strength steel benchmark frames. All files and data are labelled in relation to the eight frame specimens: HSS-I-65×116-V, HSS-I-65×116-H, HSS-I-65×116-V&H-1, HSS-I-65×116-V&H-2, HSS-I-80×136-V, HSS-I-80×136-H, HSS-I-80×136-V&H-1, HSS-I-80×136-V&H-2. The HSS-I designates a welded I-section made of S690 steel, followed by the nominal dimensions of the I-section in millimeters and finally the load case. Two cross-section dimensions were considered with flange width *B* × outer section depth *H* × flange thickness *t*_f_ × web thickness *t*_w_ equal to: 65×116×8×8 mm and 80×136×8×8 mm. For each tested frame, the same welded I-section was used for both the columns and beams. Four load cases were considered: vertical load *V* only applied at the mid-span of the beam, horizontal load *H* only applied to the bottom loading beam and two combinations of combined *V* and *H*.

The data repository is subdivided into two primary folders: (1) Experimental dataset and (2) Numerical dataset. In addition to the two folders, there is a summary file ‘Summary – test vs GMNIA.xlsx’ to present a comparison of the experimental and numerical results.

Within the experimental dataset folder there are data files containing i) material properties, ii) geometric properties, iii) imperfection measurements, iv) test instrumentation and v) test results. The results from the tests are presented as both raw data (from 16 strain gauges, 6 inclinometers, 5 string potentiometers and load-displacement recordings) in excel spreadsheets named according to the frame specimen, e.g. ‘HSS-I-65×116-H.xlsx’ corresponds to the results of frame HSS-I-65×116 subjected to horizontal loading only, as well as photos of the failure mode of each frame test (contained in the sub-folder ‘Failure modes’ and labelled according to the frame specimen e.g. ‘HSS-I-80×136-V&H-1_TestFailureMode.png’ corresponds to the failure mode of frame HSS-I-80×136 subjected to the first combined vertical and horizontal load case).

Within the numerical dataset folder there are data files containing i) a summary of the frame finite element (FE) modelling details, ii) FE model Abaqus input files and iii) geometrically and materially nonlinear analysis (GMNIA) results. The summary of the FE modelling details includes all necessary data for the numerical simulation of the test specimens including the boundary conditions, local imperfections [3,4] and residual stress pattern [5,6] assumed in the models. The GMNIA results are presented as both load-displacement information in a single excel spreadsheet ‘GMNIA load-displacement curves.xlsx’, with a sheet containing the results from the GMNIA simulation for each frame specimen, as well as images of the failure mode of each GMNIA simulation (contained in the sub-folder ‘Failure modes’ and labelled according to the frame specimen e.g. ‘HSS-I-80×136-V&H-1_GMNIAFailureMode.png’ corresponds to the failure mode of the GMNIA of frame HSS-I-80×136 subjected to the first combined vertical and horizontal load case).

**Fig. 1:**
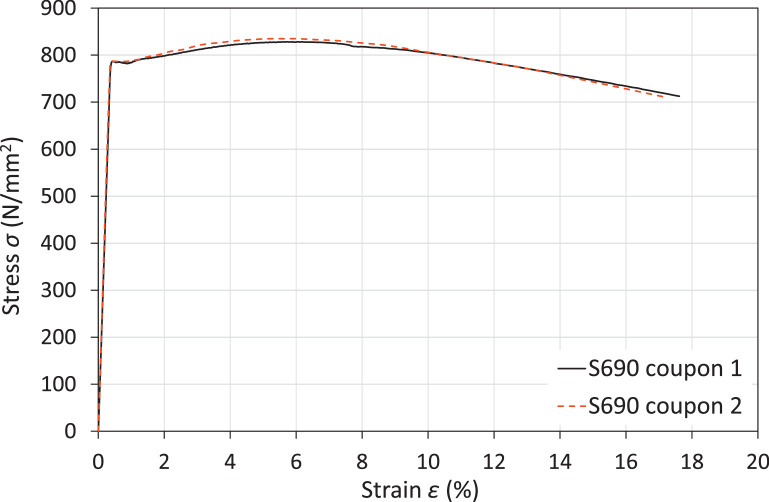
Engineering stress-strain curves obtained from tensile coupon tests for the tested frame specimens [1].

The chemical composition and summary of material properties is given in ‘Measured material properties – coupon test results.xlsx’. The raw stress-strain response from the two material coupon tests are shown in Fig. 1, with the key measured material properties given in Table 1.

**Table 1:** Key measured material properties obtained from S690 coupon tests [1].

Steel grade	*E* (N/mm^2^)	*f*_y_ (N/mm^2^)	*f*_u_ (N/mm^2^)	*ε*_sh_ (%)	*ε*_u_ (%)	*ε*_f_ (%)	*f*_u_/*f*_y_	*ε*_u_/*ε*_y_
S690	212,000	782.5	828.4	0.96	6.17	17.3	1.06	16.7

**Fig. 2:**
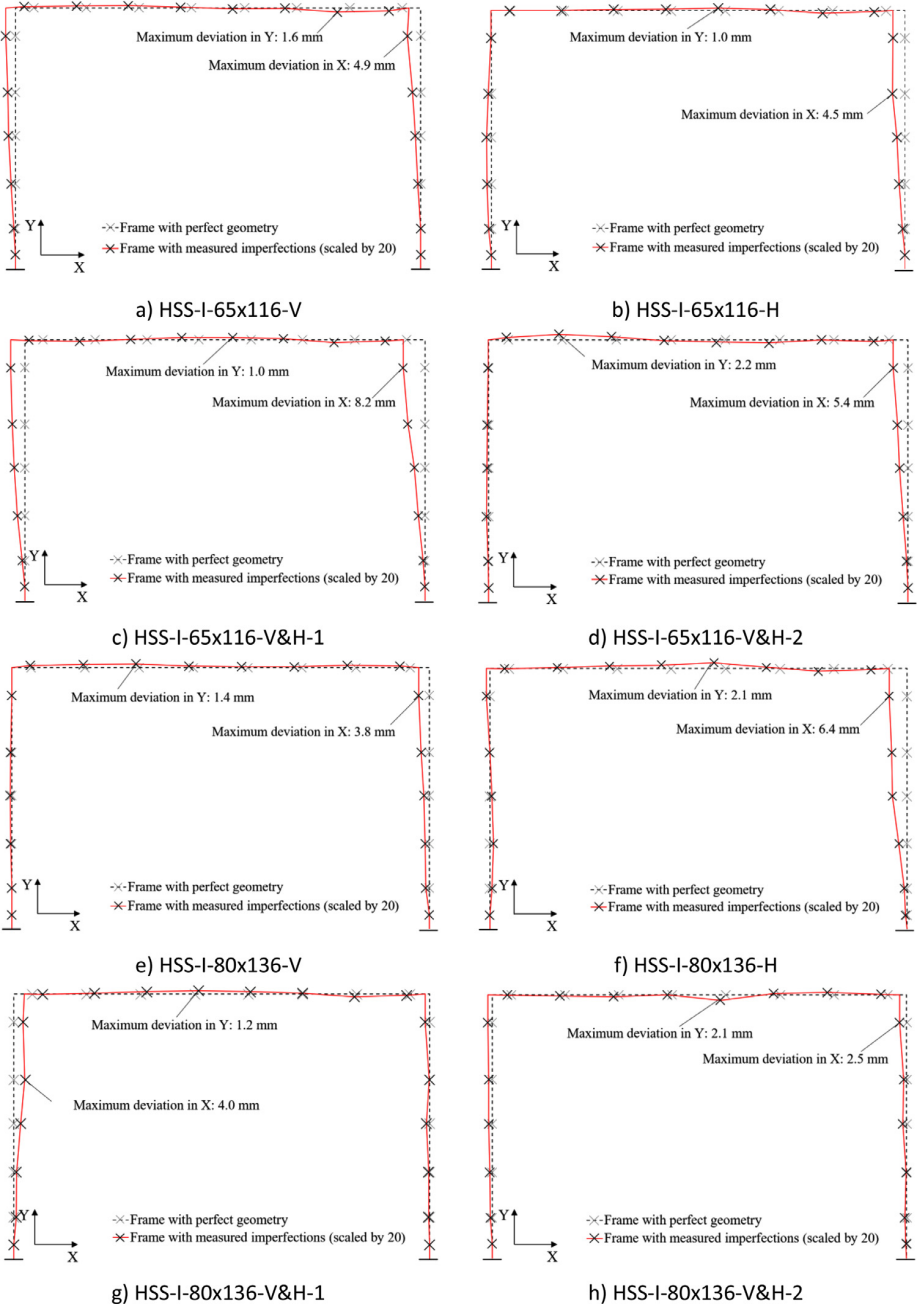
Measured in-plane geometric imperfections of tested frame specimens [1].

The geometry of the frame specimens is shown in ‘Test instrumentation.pdf’ with the data for the measured cross-sectional properties given in ‘Measured geometric properties.xlsx’. A summary of the average measured cross-sectional properties of the frame specimens is given in Table 2. The initial in-plane geometric imperfection measurements for the frames are shown in Fig. 2. These were plotted based on the raw data in ‘Measured geometric imperfections.xlsx’.

**Table 2:** Average measured cross-sectional properties of frame specimens [1].

Frame specimen	*B* (mm)	*H* (mm)	*t*_f_ (mm)	*t*_w_ (mm)	*t*_weld_ (mm)
HSS-I-65×116-V	63.64	115.20	8.31	8.21	5.21
HSS-I-65×116-H	64.10	115.73	8.41	8.29	5.52
HSS-I-65×116-V&H-1	64.08	115.79	8.36	8.22	4.96
HSS-I-65×116-V&H-2	64.00	115.30	8.33	8.24	4.87
HSS-I-80×136-V	79.06	136.77	8.47	8.39	5.61
HSS-I-80×136-H	79.63	136.46	8.51	8.42	5.56
HSS-I-80×136-V&H-1	78.20	136.76	8.36	8.26	5.11
HSS-I-80×136-V&H-2	79.02	136.60	8.35	8.28	4.92

**Fig. 3:**
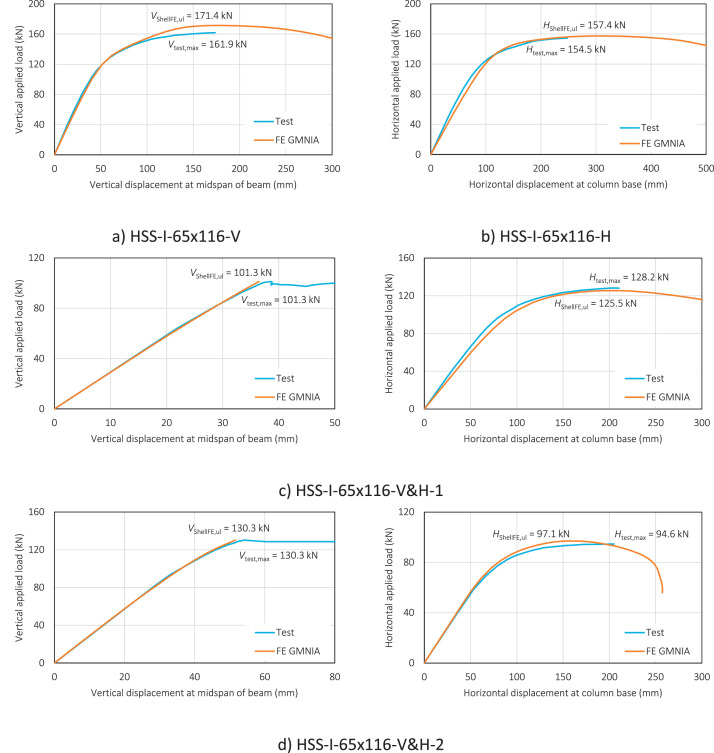
Load-displacement curves for the HSS-I-65×116 frame specimens.

**Fig. 4:**
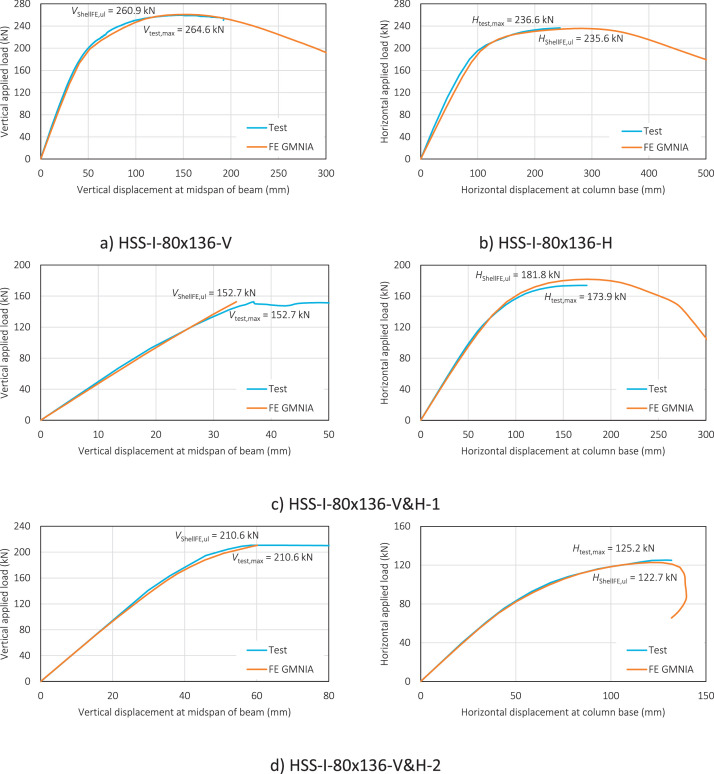
Load-displacement curves for the HSS-I-80×136 frame specimens.

The load-displacement curves in Figs. 3 and 4 (as included in ‘Summary – test vs GMNIA.xlsx’) are for both the test and FE GMNIA of the HSS-I-65×116 and HSS-I-80×136 frame specimens, respectively. These are plotted based on from the data recorded during the in-plane tests of the specimens and from the GMNIA of the FE models – corresponding to running the input files (e.g. HSS-I-65×116.inp) in Abaqus. Note that for the frame specimens subjected to combined loading (H&V-1 and H&V-2), the load-displacement curves for both load steps are presented i.e. the load-displacement curve corresponding to the vertical applied load step and the load-displacement curve for the subsequent horizontal applied load step.

**Fig. 5:**
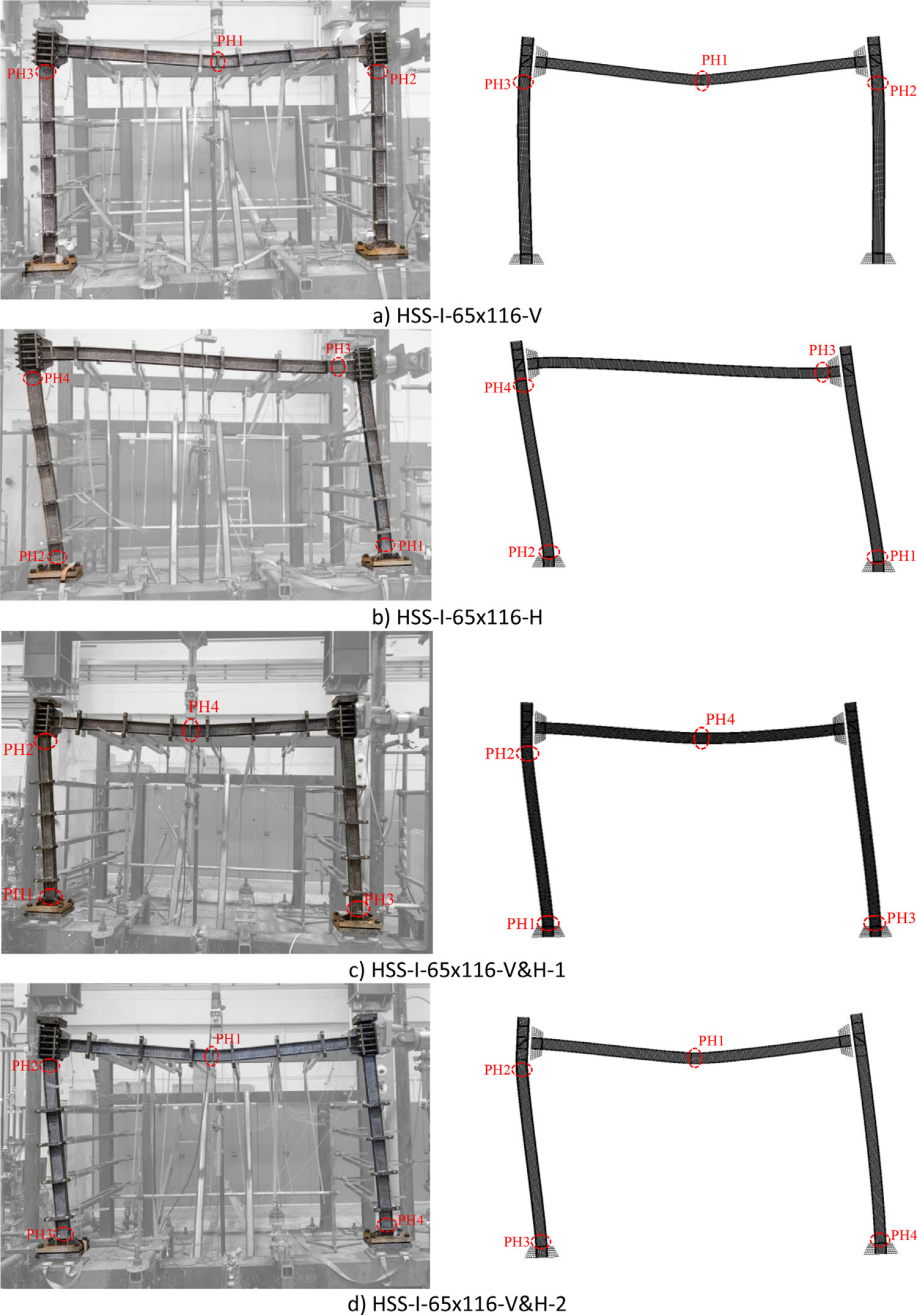
Experimental [1] and numerical failure modes of the HSS-I-65×116 frame specimens (‘PH’ represents plastic hinge, ‘1’, ‘2’, ‘3’ and ‘4’ indicate the sequence of the plastic hinge formation).

**Fig. 6:**
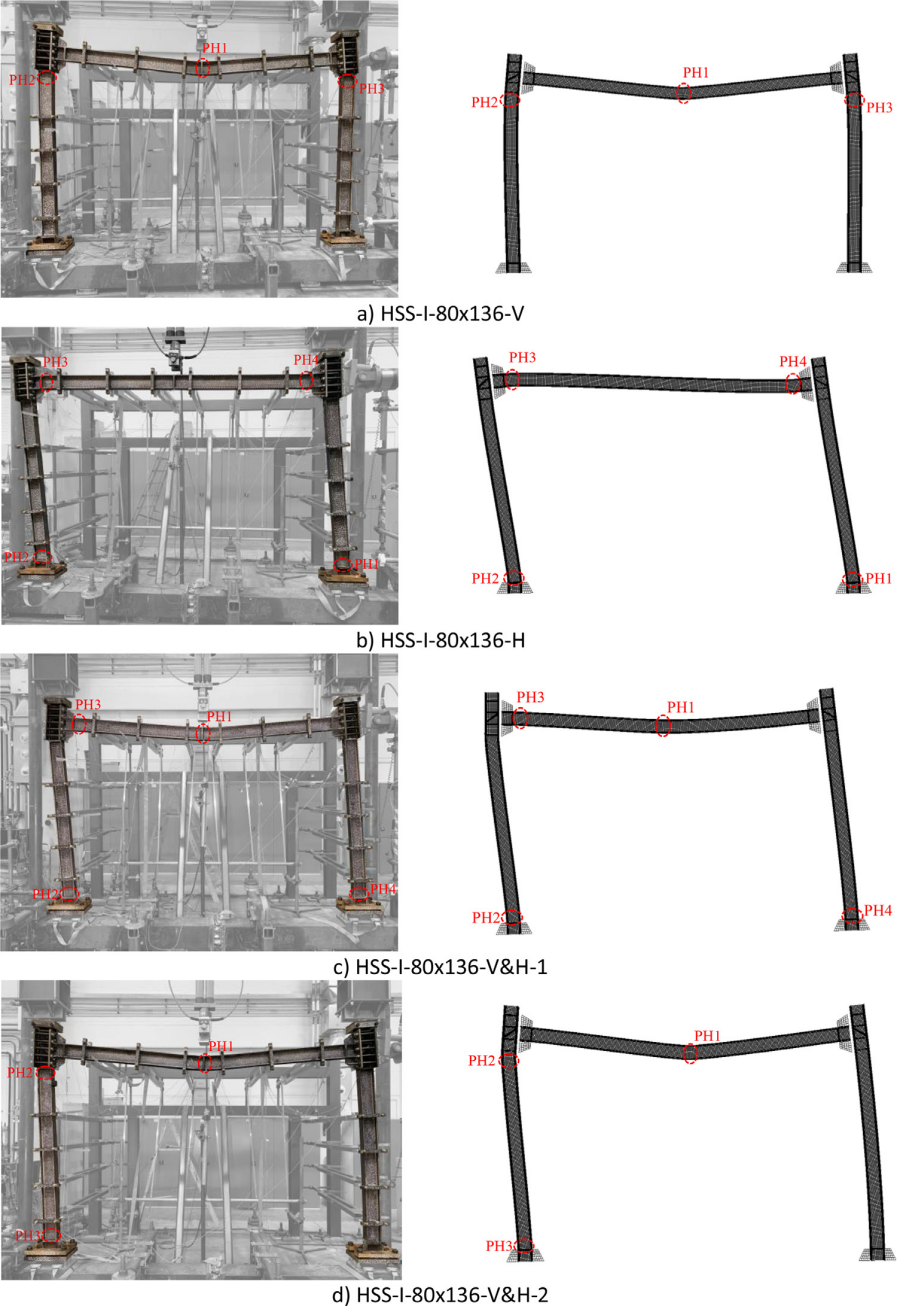
Experimental [1] and numerical failure modes of the HSS-I-80×136 frame specimens (‘PH’ represents plastic hinge, ‘1’, ‘2’, ‘3’ and ‘4’ indicate the sequence of the plastic hinge formation).

Images of the failure modes from both the tested frame specimens and comparison FE frame models are shown in Figs. 5 and 6 for the HSS-I-65×116 and HSS-I-80×136 frames, respectively, as found as .png files in the ‘Failure modes’ sub-folders in both the ‘Experimental dataset’ and ‘Numerical dataset’ folders. The sequence of the plastic hinge (PH) formation and the locations of the formed plastic hinges are also illustrated in Figs. 5 and 6.

## 3 Experimental Design, Materials and Methods

### 3.1 Experimental datasets

The material coupon tests were carried out using an Instron hydraulic 250 kN testing machine under displacement control, with a displacement rate of 0.05 mm/min up to the yield strength and a higher rate of 0.5 mm/min for the post-yield range. The longitudinal tensile strain was determined based on the average response from two strain gauges attached to the mid-height of the tensile coupons, while the stress was determined from the applied loading divided by the cross-sectional area, determined based on measurements using digital callipers. The chemical composition of the S690 material was provided by the manufacturer.

**Fig. 7:**
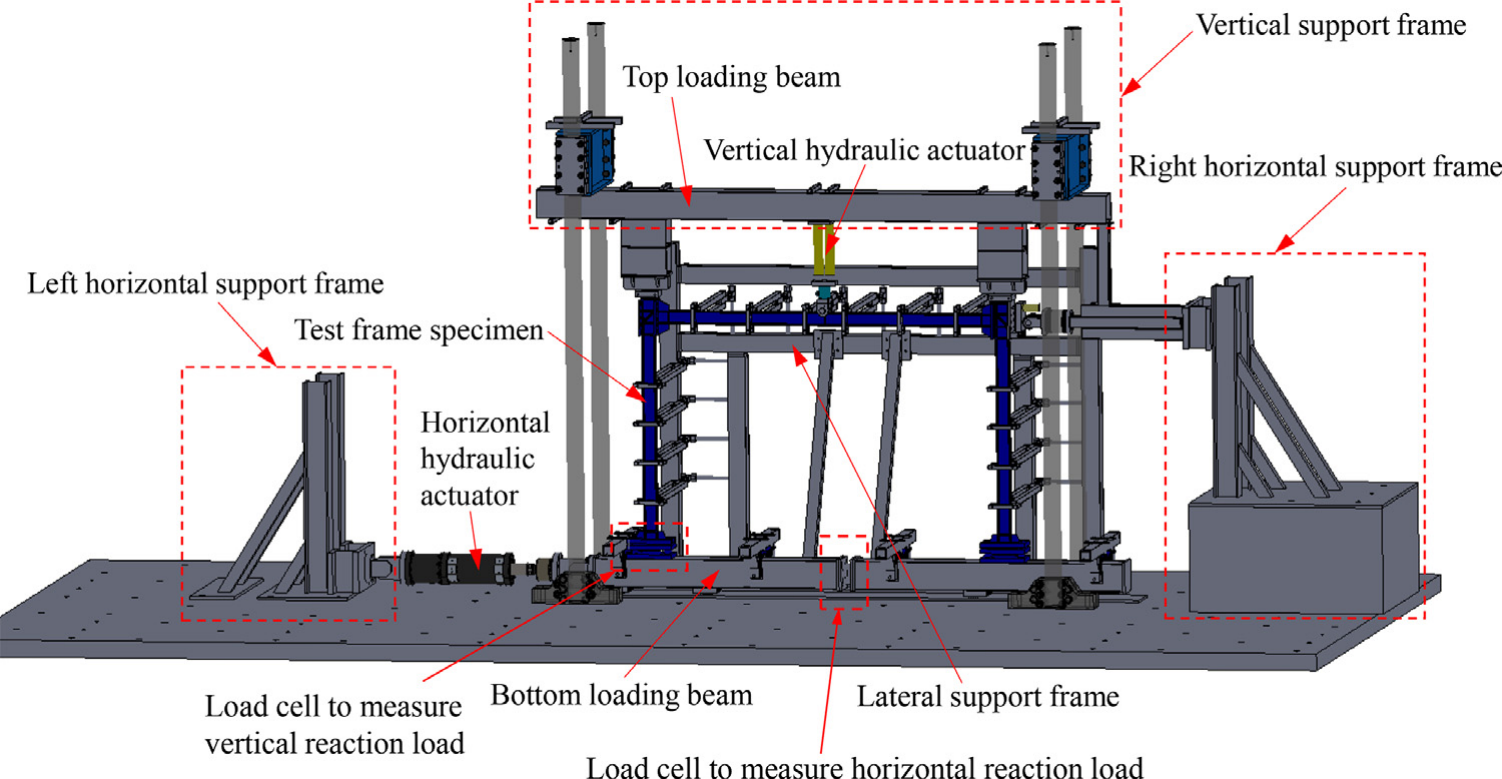
Schematic solidworks model of frame test setup [1].

For the frame tests, the vertical load was applied using two 232 kN hydraulic actuators, while the horizontal load was applied to the bottom loading beam using a 250 kN hydraulic actuator, as shown in Fig. 7. The vertical load was applied under load control at a rate of approximately 5 kN/min, while the horizontal load was introduced under displacement control at a rate of approximately 2 mm/min. For the frames subjected to combined loading, the vertical load *V*_test_ was first applied at the mid-span of the beam until the pre-determined load was reached (i.e. 100 kN and 130 kN for HSS-I-65×116-V&H-1 and HSS-I-65×116-V&H-2, respectively, and 150 kN and 210 kN for HSS-I-80×136-V&H-1 and HSS-I-80×136-V&H-2, respectively). The vertical load *V*_test_ was then held constant while the horizontal load *H*_test_ was applied at the column base through the bottom loading beam until either collapse of the frame or excessive deformation occurred.

Three fabricated load cells were employed to measure the reaction forces and moments at the column bases. Two load cells were used to measure the vertical reaction loads and bending moments at the column bases, while a third load cell was welded into the middle of the bottom loading beam to determine the horizontal reaction loads (i.e. shear forces) at the column bases, as shown in Fig. 7. Each of the load cells consisted of two 38 mm-thick steel plates and four cylindrical steel studs (80 mm in depth and 50 mm in diameter) welded in between the two plates. Each of the four steel studs was equipped with four strain gauges, to measure the longitudinal strains at different locations; these strains were used to determine the applied load and bending moment.

**Fig. 8:**
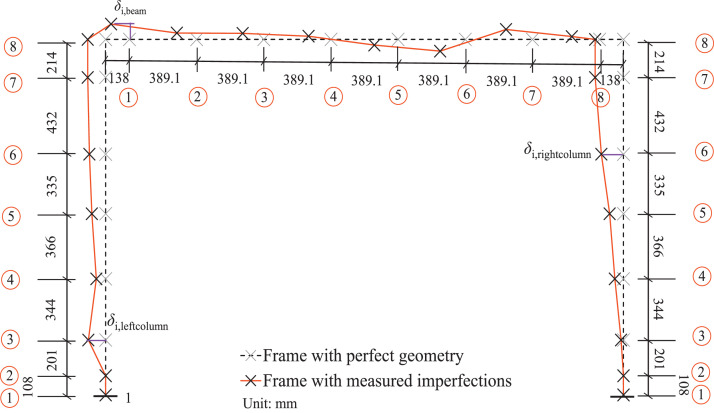
Illustration of initial geometric imperfection measurements taken around each frame specimen for determination of initial out-of-plumbness and member out-of-straightness.

The initial geometric imperfections, including out-of-plumbness and out-of-straightness, of the frame specimens were measured at eight locations along each member, as shown in Fig. 8. Plumb and horizontal lines were generated using a laser adjacent to the columns and beams of the frame specimens, respectively, to serve as reference lines from which deviations could be measured at the different locations, as marked with cross-shaped points in Fig. 8.

**Fig. 9:**
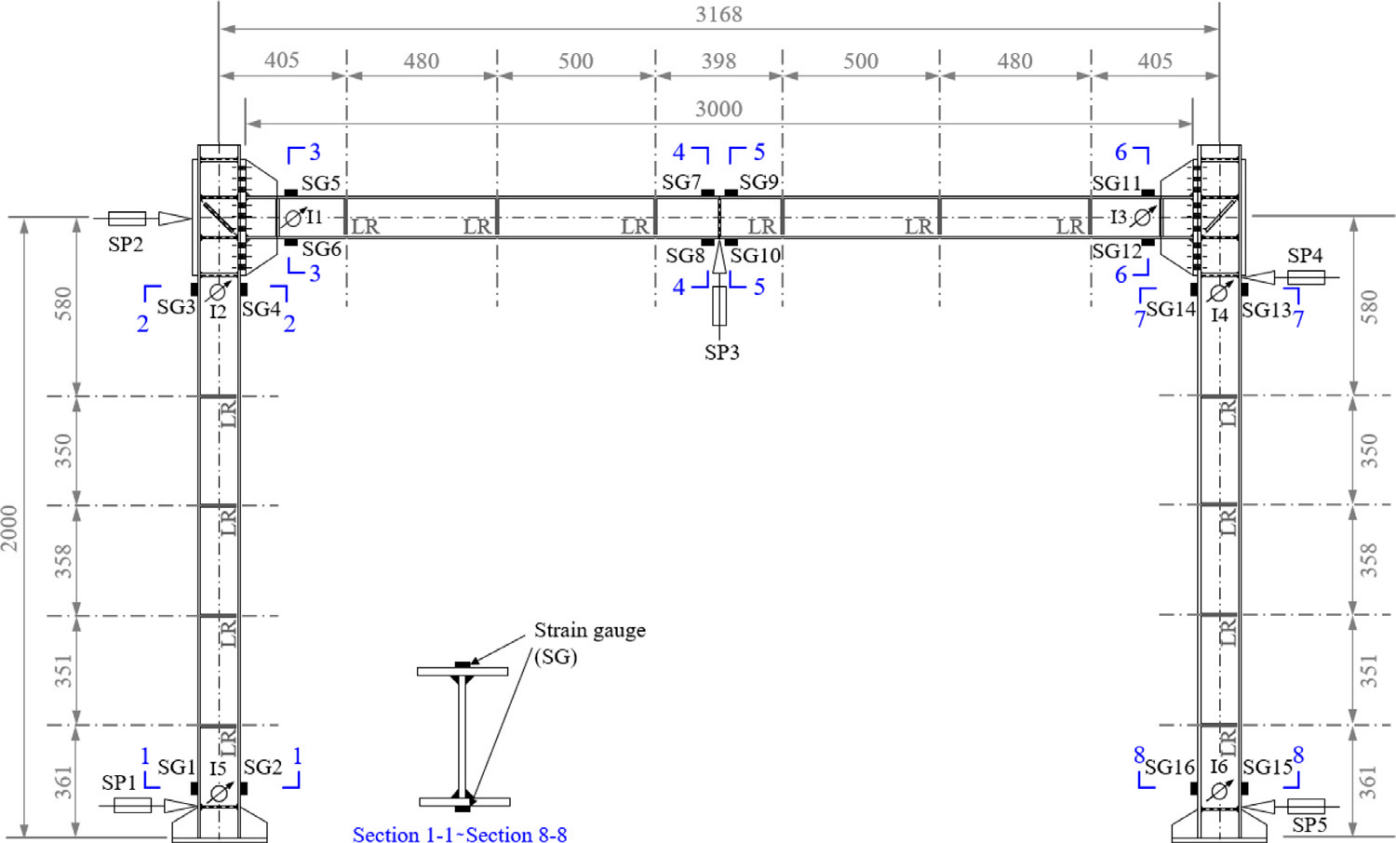
Configuration of frame specimens and instrumentation, including strain gauges (SG), string potentiometers (SP), inclinometers (I) and lateral restraints (LR) [1]. Dimensions are shown in mm.

A bespoke restraint system was designed to prevent out-of-plane instability of the test frames without inducing any undesirable in-plane restraint. The restraint system comprised 14 out-of-plane lateral restraints (LR), with 6 used to provide lateral restraint to the beam and 4 used to provide lateral restraint to each column, as shown in Fig. 9.

Electrical resistance strain gauges (SG) were mounted on the top and bottom flanges at eight locations, as shown in Fig. 9, on the frame specimens to allow for estimation of bending moments and to monitor the strain development histories during testing. String potentiometers (SP) were used to measure the in-plane vertical deflections at the mid-span of the beam (SP3 in Fig. 9) and the in-plane horizontal deflections at the base and top of each column (SP1, SP2, SP4 and SP5 in Fig. 9). Finally, a total of six inclinometers (I) were employed on each frame to measure the rotation of the connections and to evaluate their rotational stiffnesses.

### 3.2 Numerical datasets

Geometrically and materially nonlinear analysis using shell (element type S4R [7], as also successfully employed in [8,9]) FE models with imperfections were developed to simulate the response of the eight tested frame specimens in the general purpose FE software Abaqus. The measured dimensions, cross-section geometry, material properties and member and sway imperfection amplitudes were used as input parameters for the FE models. Geometric imperfections were introduced into the developed shell FE models by modifying the nodal coordinates of the perfect geometry. The measured engineering stress-strain curves from the S690 steel coupon tests were incorporated into the FE models. To account for the change in cross-sectional geometries under load using the shell elements, the measured engineering stress-strain curves were converted to the true stress-plastic strain relationships.

**Fig. 10:**
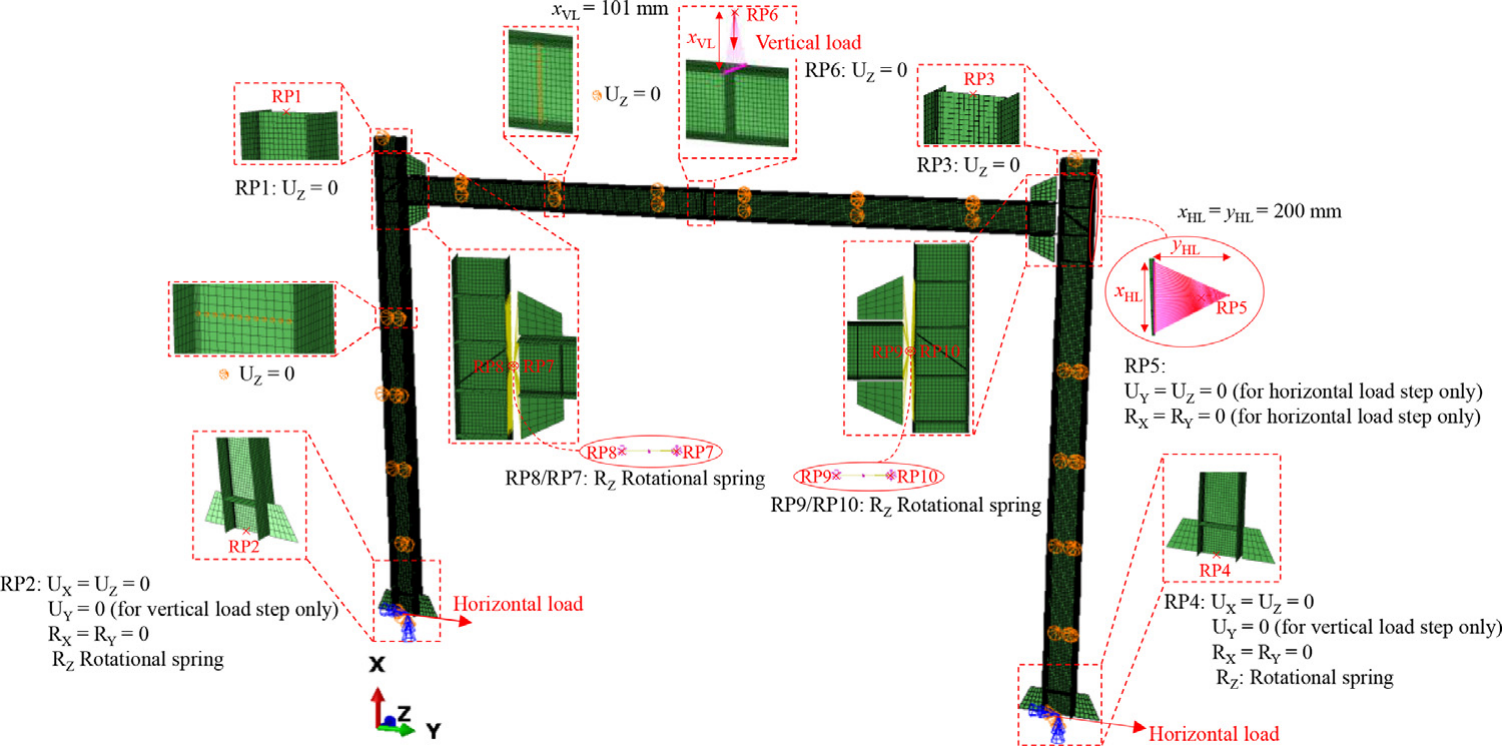
Boundary conditions employed in shell FE models.

The stiffeners employed in the test frames were modelled using the same shell element S4R as employed for the frame structural members. The stiffeners were assumed to be rigidly connected to their corresponding plates using *TIE constraints, and the true stress-plastic strain data derived from the measured engineering stress-strain curves of the S690 steel were employed to define the material properties of all stiffeners. To simulate the boundary conditions employed in the tests, each end section of the column members of the frame model was coupled to a reference point (RP) located at the centroid of the corresponding cross-section through kinematic coupling constraints, with suitable boundary conditions in accordance with the test setup applied to these reference points, as shown in Fig. 10. The out-of-plane displacement degree of freedom (i.e. *U*_Z_) was restrained at the top section of each column, where lateral restraints were provided in the frame test setup. All degrees of freedom apart from the rotation about the cross-section major axis (i.e. Z-axis, see Fig. 10) were restrained at the bottom section of each column during the application of the vertical load (if any), while the degree of freedom corresponding to the horizontal in-plane displacement (i.e. *U*_Y_) was released (or set to zero) when the horizontal load (if any) started to be applied at the bottom reference points. The semi-rigid nature of the column base connections was modelled by positioning an ABAQUS SPRING1 element between the reference point at each column end section (i.e. RP2 and RP4) and the ground, with its rotational stiffness about the cross-section major axis taken as the value measured from the corresponding frame experiment, as provided in ‘Summary of test results – Tables.xlsx’. Similarly, each semi-rigid beam-to-column connection of the frame specimens was modelled by a linear rotational spring (using the ABAQUS SPRING2 element) connecting two reference points, which were coupled to the nodes at the corresponding end section of the beam and at the relevant area of the flange of the corresponding column, as illustrated in Fig. 10; the SPRING2 element employed in the frame FE model acted in the rotational direction about the cross-section major axis with a stiffness equal to the corresponding experimentally measured value (as provided in ‘Summary of test results – Tables.xlsx’). The out-of-plane displacement degree of freedom (i.e. *U*_Z_) of the nodes located at the web of the beam and columns of the frame FE models, in positions corresponding to the location of the lateral restraints in the frame test setup (see Fig. 9), was constrained.

The vertical load was applied to a reference point (RF6 in Fig. 10) which was positioned at the centroid of the vertical actuator and coupled to nodes at the mid-span top flange of the frame beam as shown in Fig. 10. Note that a web stiffener was also included in the shell FE models at the mid-span of the beam, where the vertical load was applied, to prevent localised web crippling failure. The same value of in-plane horizontal displacement (i.e. *U*_Y_) was applied at the two reference points at the bottom end section of the two columns (i.e. RP2 and RP4) to mimic the displacement-controlled horizontal loading scheme adopted in the frame experiments. The horizontal restraint at the top of the right-hand column for frame specimens subjected to horizontal loading was modelled by assigning suitable boundary conditions to a reference point (RP5 in Fig. 10), which was located at the centroid of the strengthening plate region of the right hand beam-to-column connection.

For the frames subjected to either vertical load only or horizontal load only, only one corresponding analysis step was established and the geometrically and materially nonlinear analyses with imperfections (GMNIA) were solved using the modified Riks arc-length method in order to trace the full load-displacement response of the frames. For the frames subjected to the combined loading, two steps – the first corresponding to the vertical load and the second to the horizontal load – were included and the GMNIA were solved using the General Static method and the modified Riks arc-length method for the first and second steps, respectively. The load-displacement data are extracted from the odb file generated from running each input file in Abaqus. Both the load and displacement data are extracted at unique nodal locations corresponding to the predefined reference points in the FE models.

## Limitations

Not applicable.

## Ethics Statement

This manuscript adheres to ethics in publishing standards.

## CRediT authorship contribution statement

**Fiona Walport:** Data curation, Visualization, Writing – original draft. **Yufei Zhu:** Data curation, Software, Validation, Formal analysis, Visualization, Investigation, Writing – review & editing. **Xiang Yun:** Conceptualization, Formal analysis, Methodology, Data curation, Investigation, Writing – review & editing. **Leroy Gardner:** Conceptualization, Methodology, Investigation, Resources, Funding acquisition, Supervision, Writing – review & editing.

## Data Availability

Structural tests on high strength steel welded I-section frames (Original data) (Mendeley Data)
